# LRRK2 phosphorylates pre-synaptic N-ethylmaleimide sensitive fusion (NSF) protein enhancing its ATPase activity and SNARE complex disassembling rate

**DOI:** 10.1186/s13024-015-0066-z

**Published:** 2016-01-13

**Authors:** Elisa Belluzzi, Adriano Gonnelli, Maria-Daniela Cirnaru, Antonella Marte, Nicoletta Plotegher, Isabella Russo, Laura Civiero, Susanna Cogo, Maria Perèz Carrion, Cinzia Franchin, Giorgio Arrigoni, Mariano Beltramini, Luigi Bubacco, Franco Onofri, Giovanni Piccoli, Elisa Greggio

**Affiliations:** Department of Biology, University of Padova, via Ugo Bassi 58/B, 35131 Padova, Italy; San Raffaele Scientific Park & University Vita-Salute San Raffaele, Milan, Italy; Department of Experimental Medicine, University of Genova, Genova, Italy; Department of Biomedical Sciences, University of Padova, Padova, Italy; Proteomics Center of Padova University, Padova, Italy; IN-CNR Milano, Milano, Italy; Present Address: Department of Cell and Developmental Biology, University College London, London, WC1E 6BT UK; Present Address: Rheumatology Unit, Department of Medicine – DIMED, University Hospital of Padova, Padova, Italy

**Keywords:** Parkinson’s disease, Leucine-rich repeat kinase 2, N-ethylmaleimide sensitive fusion, Presynapse, Phosphorylation

## Abstract

**Background:**

*Lrrk2*, a gene linked to Parkinson’s disease, encodes a large scaffolding protein with kinase and GTPase activities implicated in vesicle and cytoskeletal-related processes. At the presynaptic site, LRRK2 associates with synaptic vesicles through interaction with a panel of presynaptic proteins.

**Results:**

Here, we show that LRRK2 kinase activity influences the dynamics of synaptic vesicle fusion. We therefore investigated whether LRRK2 phosphorylates component(s) of the exo/endocytosis machinery. We have previously observed that LRRK2 interacts with NSF, a hexameric AAA+ ATPase that couples ATP hydrolysis to the disassembling of SNARE proteins allowing them to enter another fusion cycle during synaptic exocytosis. Here, we demonstrate that NSF is a substrate of LRRK2 kinase activity. LRRK2 phosphorylates full-length NSF at threonine 645 in the ATP binding pocket of D2 domain. Functionally, NSF phosphorylated by LRRK2 displays enhanced ATPase activity and increased rate of SNARE complex disassembling. Substitution of threonine 645 with alanine abrogates LRRK2-mediated increased ATPase activity.

**Conclusions:**

Given that the most common Parkinson’s disease LRRK2 G2019S mutation displays increased kinase activity, our results suggest that mutant LRRK2 may impair synaptic vesicle dynamics *via* aberrant phosphorylation of NSF.

**Electronic supplementary material:**

The online version of this article (doi:10.1186/s13024-015-0066-z) contains supplementary material, which is available to authorized users.

## Background

Leucine-rich repeat kinase 2 (LRRK2) is a large kinase with protein-to-protein interaction domains and dual enzymatic activities. The catalytic core includes a ROC (Ras Of Complex) domain with GTPase activity, followed by a COR (C-terminus Of ROC) domain likely involved in protein dimerization, and a serine-threonine kinase domain [1–3]. Mutations in *Lrrk2* cause late-onset autosomal dominant Parkinson’s disease (PD) [4, 5], whereas more common variants around the *Lrrk2* locus act as risk factors for disease [6, 7]. As the most common G2019S mutation increases kinase activity in vitro and in vivo by ~3 fold, LRRK2 is being intensively explored as a pharmacological target for the treatment of PD [8]. Several substrates of LRRK2’s kinase activity have been reported, however few of these have been extensively validated at a physiological level [9]. There is, therefore, an increasing interest in identifying LRRK2 substrates and cellular pathways compromised during pathological conditions that could serve as therapeutic alternatives to directly targeting LRRK2 kinase activity. LRRK2 has been found associated with various membrane structures, including synaptic vesicles (SV) [10–15]. Multiple studies on different experimental models support a role for LRRK2 at the synapse. Mutant LRRK2 rodent models display defects in neurotransmission [16–19], and LRRK2 overexpression or knockdown results in impaired SV endocytosis/exocytosis [15, 20]. We recently showed that LRRK2 binds SV *via* interaction with a number of presynaptic proteins [21] and that its kinase activity modulates these interactions and impacts on SV dynamics [22]. Among the LRRK2 interactors identified, we found N-ethylmaleimide sensitive factor (NSF), which is involved in the fusion of SV orchestrated by SNARE (Soluble NSF-Attachment protein REceptor) proteins. During membrane fusion, vesicular and target SNAREs assemble into an alpha-helical trans-SNARE complex that juxtaposes the two membranes together to catalyze membrane fusion. NSF is the ATPase that catalyzes the release of SNARE complexes, thus allowing SV endocytosis and the next cycle of fusion [23]. Notably, NSF activity is tightly controlled by phosphorylation/dephosphorylation [24–26]. In the present study, using dynamic assays of SV cycling, we found that SV fusion is altered by LRRK2 kinase function, suggesting components of the exo/endocytic machinery may be a target of LRRK2 kinase activity. Given that LRRK2 interacts with NSF, we assessed whether NSF is a substrate for LRRK2 kinase activity. We found that LRRK2 can efficiently phosphorylate NSF in vitro, with phosphorylation primarily occurring at T645. Importantly, phosphorylated NSF displays enhanced ATPase activity and increased rate of SNARE complex disassembling in vitro. Our data implicate LRRK2 kinase activity in the regulation of SV exo/endocytosis by phospho-modulation of NSF activity and suggest that pathological LRRK2 may disturb SV dynamics *via* aberrant phosphorylation of NSF.

## Results

### LRRK2 kinase activity influences synaptic vesicle dynamics

**Fig. 1:**
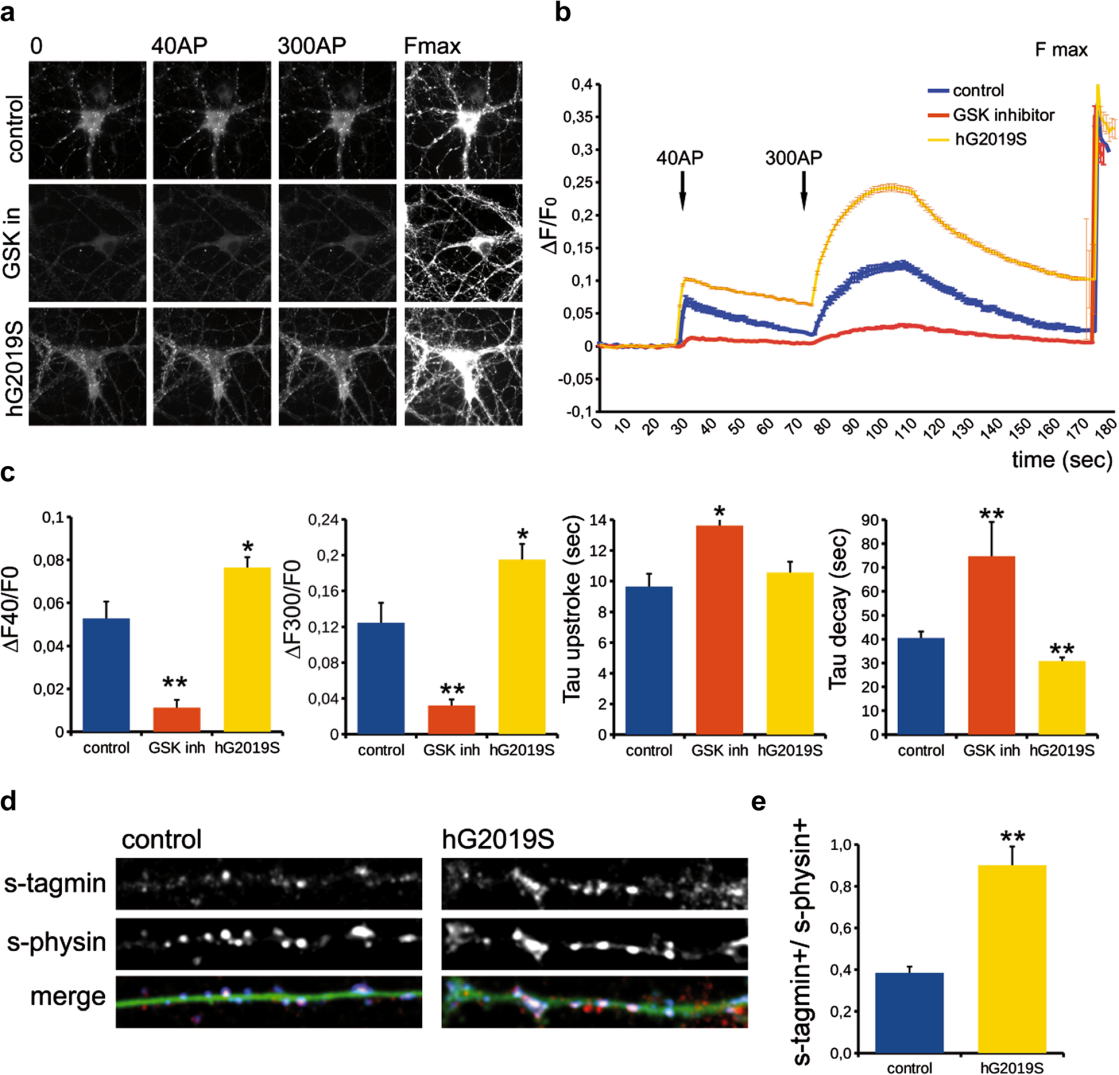
LRRK2 kinase activity modulates synaptic vesicle fusion. **a** We recorded synaptophluorin fluorescence from DIV14 wild-type cortical neurons treated with DMSO (control) or treated with LRRK2 inhibitor GSK2578215A (GSK in, 0.2 μM, 2 h) and from cortical neurons obtained from BAC hG2019S mice (hG2019S). Representative snapshots were taken at DIV16 from 1 Hz recordings at rest (0), after 40 action potential stimulation (40AP), after 300 action potential stimulation (300AP) and upon neutralization with 50 mM NH_4_Cl to reveal total fluorescence (Fmax). Panels size is 113 × 113 μm. **b** The graph shows representative pattern of fluorescence. Y-axis reports ΔF/F0 at the given time point (second). **c** The graphs report the increase in fluorescence after 40AP (ΔF40/F0) and 300AP (ΔF300/F0) and the kinetic of signal after 300AP expressed as time constant describing the increase (tau upstroke) and decay (tau decay) of fluorescence. Data are expressed as mean ± SEM, *n* = 4, at least 50 boutons from minimum five neurons were analyzed for experiment (* *p* < 0.05; ** *p* < 0.01 versus control, ANOVA). **d** The exo/endocytotic assay was performed at DIV14 on wild-type and BAC hG2019S cortical neurons infected at DIV4 with virus expressing GFP. Cycling SV appears as synaptotagmin (s-tagmin) positive clusters along neuron processes. Total SV pool was revealed by staining with anti-synaptophysin antibodies upon fixation and permeabilization. Images show signals acquired for synaptotagmin, synaptophysin and their superimposition plus GFP (merge). Panel size is 28 × 4 μm. (e) The percentage of s-tagmin and s-physin positive clusters within the totality of s-physin positive clusters reflects the pool of cycling vesicles. Data are expressed as mean ± SEM, *n* = 4, at least 7 neurons were analyzed for experiment (** *p* < 0.01 Student’s *t*-test)

We recently reported that inhibition of LRRK2 kinase activity causes impairment in synaptic vesicles (SV) dynamics, indicating a role for LRRK2 catalytic activity in SV fusion cycle [22]. To further determine the role of LRRK2 kinase activity at the presynapse, we performed dynamic assays of SV taking advantage of the sypHy assay in two complementary models: a) primary cortical cultures in the presence or absence of the LRRK2 inhibitor GSK2578215A (GSK in, 0.2 μM, 2 h), a brain penetrant, selective LRRK2 inhibitor (IC_50_ 10 nM) [27]; b) primary cortical neurons obtained from BAC hG2019S mice characterized by higher LRRK2 kinase activity [28]. GSK treatment induced LRRK2 dephosphorylation at Ser935, as predicted (Additional file 1: Figure S1-b), but did not cause protein destabilization (Additional file 1: Figure S1a–c), whereas BAC hG2019S neurons displayed increased LRRK2 expression due to the presence of the transgene (Additional file 1: Figure S1a-b-c). Synaptophysin-pHluorin (sypHy) is a pH-sensitive fluorescent reporter that, by analogy with the original synaptopHluorin (synaptobrevin-pHluorin), is quenched in the acidic intracellular space of the SV and will only become fluorescent upon SV fusion, when the contents of the SV is exposed to the more basic pH of the extracellular space [29]. As shown in Fig. 1, at the onset of the stimulus, exocytosis caused a rapid increase in sypHy fluorescence, which after cessation of the stimulus, slowly returned to baseline (Fig. 1a–b). The first stimulus, 40 AP, is predicted to mobilize SV belonging to the ready releasable pool, while 300 AP is sufficient to trigger the fusion of SV belonging to the total recycling pool [29]. Furthermore, the kinetics describing the on-set and the decay of the fluorescence are correlated to the efficiency of the exocytotic and endocytotic mechanism, respectively [29]. Interestingly, while we measured a significant impairment of SV fusion (decreased fluorescence) in the presence of GSK upon either 40 or 300 AP stimuli, BAC hG2019S neurons were characterized by a higher answer following the two stimulations (ΔF40/F0 and ΔF300/F0 respectively, Fig. 1c). Furthermore, while upon acute pharmacological inhibition the time taken for fluorescence decay was extended, τ decay was decreased in hG2019S cells (Tau decay, Fig. 1c). To further assess a role for LRRK2 in the SV cycle, we took advantage of the exo/endocytic assay previously reported [20, 21]. Using this approach, we previously demonstrated that acute pharmacological blockade of LRRK2 kinase activity impairs SV recycling [22]. Building upon these data, BAC hG2019S cortical cultures were transduced at DIV4 with control viruses co-expressing GFP to track neuronal processes and assayed at DIV14. Briefly, we exposed living cultures to rabbit polyclonal antibodies directed against the intravesicular domain of synaptotagmin1, which are internalized inside the vesicle lumen upon SV recycling [30]. Vesicles within GFP positive processes were then monitored *via* laser confocal microscopy. The vesicles appeared as clusters either synaptotagmin and synaptophysin positive (i.e. cycling vesicles) or only synaptophysin positive (Fig. 1d). The analysis showed that BAC hG2019S cultures are characterized by a significant increase in the number of synaptotagmin and synaptophysin positive clusters (Fig. 1e). The total number of synaptic contacts, however, remained unaltered despite any pharmacological treatments (Additional file 1: Figure S1d). Taken together, these results indicate that LRRK2 kinase activity is involved in the regulation of SV fusion.

### LRRK2 interacts with NSF

Having found that LRRK2 kinase activity influences SV fusion, we next asked what the molecular mechanisms behind this phenotype might be. We had previously demonstrated that LRRK2 interacts with the vesicle fusing ATPase NSF through its WD40 domain [21].

**Fig. 2:**
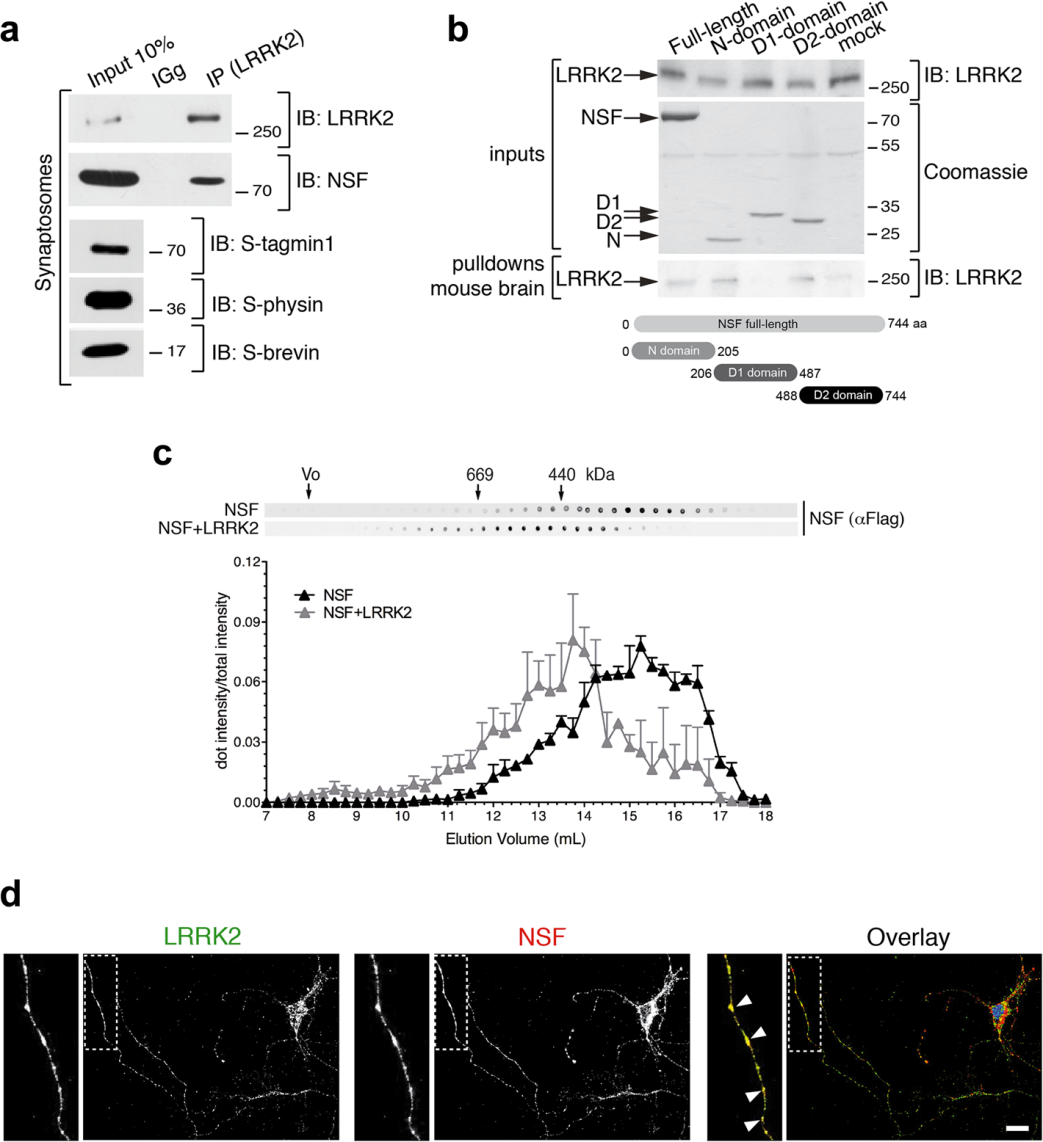
LRRK2 interacts with NSF. **a** Extracts of purified cortical synaptosomes were incubated with anti-LRRK2 antibodies or rabbit IgG. The immunocomplexes were sedimented with protein G-Sepharose and the samples were resolved by SDS-PAGE and analyzed by immunoblotting with anti NSF and anti LRRK2 antibodies. Immunoblotting against synaptotagmin 1 (S-tagmin1), synaptophysin (S-physin) and synaptobrevin (S-brevin) were performed to confirm purity of synaptosomal preparation. **b** Flag-NSF full-length or domains (N, D1, D2) purified from HEK293T and bound to M2 flag resin were incubated with a mouse brain lysate. Samples were subjected to immunoblotting using anti-LRRK2 (MJFF2) or stained with Coomassie to show flag inputs. **c** Size exclusion chromatography fractions of HEK293T expressing ectopic flag-NSF alone or together with 2xMyc-LRRK2 spotted onto nitrocellulose membrane and probed with anti-flag antibody (*n* = 3 independent experiments). **d** Immunofluorescence of primary cortical neurons stained for endogenous LRRK2 and endogenous NSF (scale bar is 10 μm)

We first confirmed LRRK2 and NSF interaction at the endogenous level in synaptosomal preparations. Using co-immunoprecipitation with endogenous proteins from rat synaptosomes, we observed that LRRK2 efficiently co-precipitates NSF (Fig. 2a). Next, we dissected the domain(s) of NSF involved in binding LRRK2. To this aim, we cloned human full-length NSF (aa 1–744) and its different domains, namely N domain (aa 1–205), D1 domain (aa 206–487) and D2 domain (aa 488–744) in fusion with a N-terminal Flag-tag and purified the proteins from HEK293T cells. Proteins bounds to Flag-conjugated beads were adjusted to equal molar concentrations and subsequently incubated with a mouse brain lysate. As shown in Fig. 2b, full-length NSF pulls-down endogenous LRRK2. Interestingly, N and D2 domains, but not D1, also pull-down endogenous LRRK2 (Fig. 2b). To further confirm the interaction between LRRK2 and NSF, we used size exclusion chromatography (SEC) to fractionate HEK293T lysates expressing Flag-NSF or co-expressing Flag-NSF and 2xMyc-LRRK2, followed by dot blot analysis. As shown in Fig. 2c, NSF elutes between 11 and 17.5 mL. Interestingly, in the presence of LRRK2, NSF elution profile shifts toward shorter retention times (elution peak between 10 and 16.5 mL) suggesting the formation of a complex with higher molecular weight than NSF alone (Fig. 2c). We also evaluated the cellular localization of endogenous NSF and LRRK2 in primary neuronal cultures and found that the two proteins largely co-localize, and co-localization is enriched within clusters along the neurites (Fig. 2d). Collectively these data indicate that LRRK2 and NSF form a complex in the cell.

### LRRK2 phosphorylates NSF in D2 domain

LRRK2 affects SV dynamics *via* its kinase activity (Fig. 1a–c) [22]. Therefore, we hypothesized that NSF could be a substrate of LRRK2 and, as such, be involved in the LRRK2 kinase dependent regulation of SV. To test this hypothesis, we performed in vitro kinase assays using recombinant LRRK2 and NSF purified from mammalian cells. We first validated recombinant human NSF biochemically. NSF purified as described in the methods section folds into hexamers when loaded with 1 mM ATP as evidenced by negative-stain transmission electron microscopy (TEM) (Additional file 1: Figure S2a). Of interest, Flag-tagged NSF purified with Flag affinity resin co-precipitates endogenous NSF as indicated by the presence of a band corresponding to endogenous NSF (Additional file 1: Figure S2b), further supporting the notion that Flag-NSF forms oligomers. To verify that purified NSF is functional, we measured ATP to ADP hydrolysis rate by isocratic reverse-phase HPLC (Additional file 1: Figure S2c-d) and malachite green colorimetric assay (Additional file 1: Figure S2e). NSF efficiently hydrolyzes ATP to ADP over time under these purification and assay conditions.

**Fig. 3:**
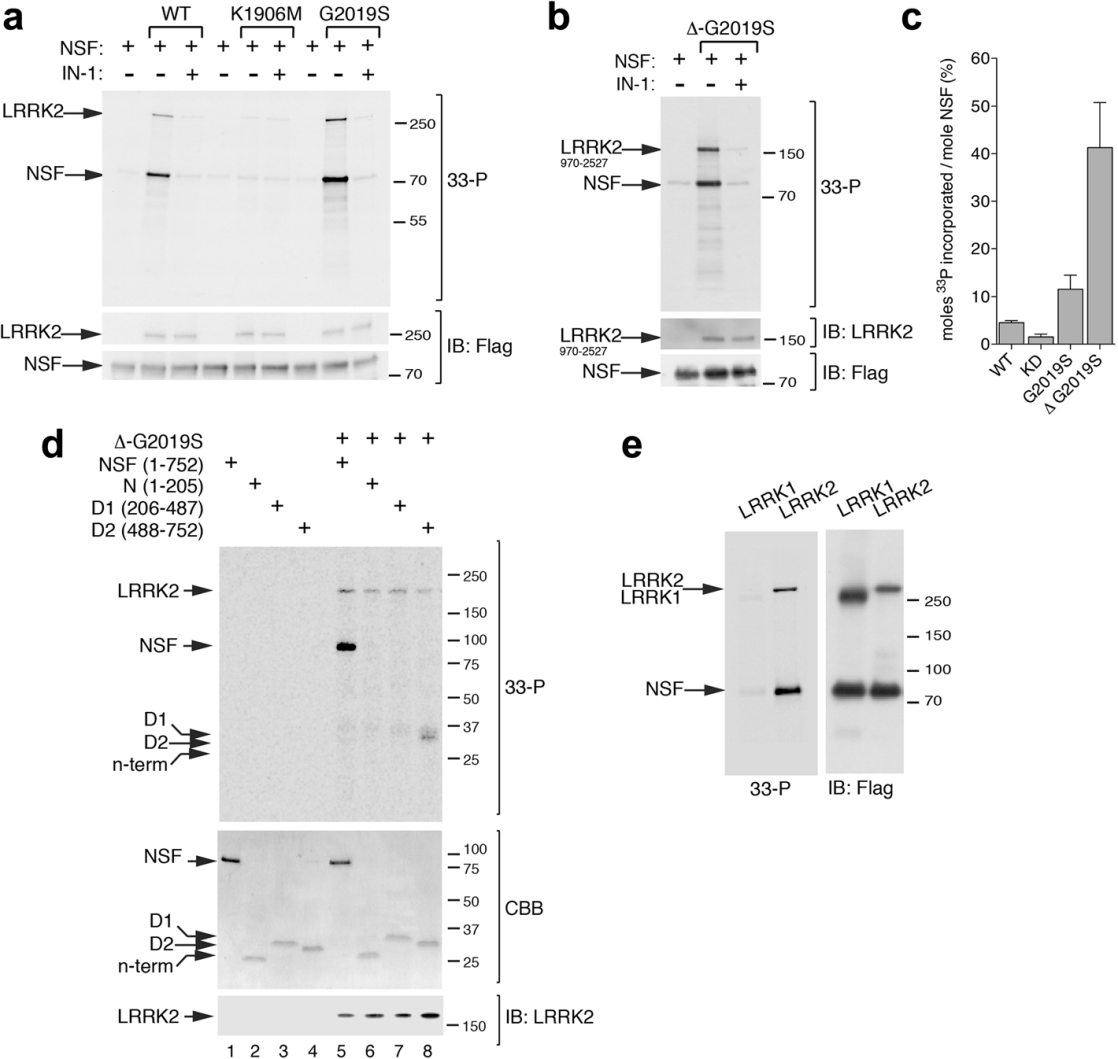
LRRK2 phosphorylates NSF. **a** In vitro radioactive kinase assays of 3x-Flag LRRK2 wild-type, K1906M (kinase dead) and G2019S (hyperactive) and flag-NSF purified from HEK293T cells at 1:10 ratio. Radioactivity incorporated was revealed by autoradiography (*upper panel*) and total proteins loaded by flag immunoblotting (*lower panels*). LRRK2 inhibitor IN-1 was used at 1 μM concentration to confirm LRRK2 specific phosphorylation on NSF. **b** In vitro kinase assays as in (a) with the hyperactive GST-LRRK2^970–2527^ fragment. **c** Quantification of moles of ^33^P incorporated by NSF using a calibration curve with known concentration of ^33^P-ATP. **d** In vitro kinase assays as in (a) using NSF full-length or domains as substrates of LRRK2 GST-LRRK2^970–2527^ kinase activity at 1:10 ratio LRRK2:NSFs. Radioactivity incorporated was revealed by autoradiography (*upper panel*) and total proteins loaded by coomassie brilliant blue (CBB) staining for NSF (*middle panel*) or LRRK2 immunoblotting (*lower panel*). **e** In vitro radioactive kinase assays of 3xFlag-LRRK1 or 3xFlag-LRRK2 and Flag-NSF as substrate at 1:10 ratio. Left panel is an example of autoradiography and *right panel* represents the corresponding immunoblot of total loading

Having validated recombinant human full-length NSF, we next purified soluble 3xFlag-LRRK2 wild-type, the hyperactive clinical mutant G2019S and the kinase dead K1906M from HEK293T cells and subsequently incubated these purified proteins with full-length NSF in kinase assay conditions [31]. As shown in Fig. 3a, at a 1:10 ratio of LRRK2:NSF, we observe robust phosphorylation of NSF by LRRK2. Importantly, in the presence of LRRK2 K1906M or upon addition of 1 μM LRRK2 IN-1 inhibitor, NSF phosphorylation corresponds to the background levels observed for NSF alone, confirming that the incorporation of radioactive phosphate is genuinely due to LRRK2 kinase activity (Fig. 3a). We confirmed LRRK2-mediated phosphorylation of NSF using the hyperactive G2019S-LRRK2^970–2527^ fragment (Fig. 3b). The stoichiometry of phosphate incorporation, measured using a calibration curve with different concentrations of ^33^P-ATP, is approximately 0.04 moles of phosphate per mole of monomeric NSF in the presence of LRRK2 wild-type, 0.1 in the presence of G2019S and 0.4 with an artificial truncated variant characterized by higher activity, G2019S-LRRK2^970–2527^ (Fig. 3c). The low value for this stoichiometry, when taken in the context of a hexameric NSF complex, is sufficient to imply the presence of at least one phosphorylated monomer per hexamer. The reaction reached a plateau after 1-hour incubation (Additional file 1: Figure S3a-b), likely due to inactivation of LRRK2 under assay conditions as previously reported [32].

To define the region(s) of NSF phosphorylated by LRRK2, we performed kinase assays in the presence of NSF full-length or isolated domains. While we failed to detect any phosphorylation when N and D1 domains were incubated with G2019S-LRRK2^970–2527^, we were able to measure phosphate incorporation in the D2 domain (Fig. 3d). Importantly, NSF is not a substrate of the cognate protein LRRK1 under these assay conditions (Fig. 3e), suggesting that this phosphorylation event is specific to LRRK2. *In toto*, these results indicate that LRRK2 likely phosphorylates the D2 domain of NSF.

### LRRK2 phosphorylates NSF at threonine 645

**Fig. 4:**
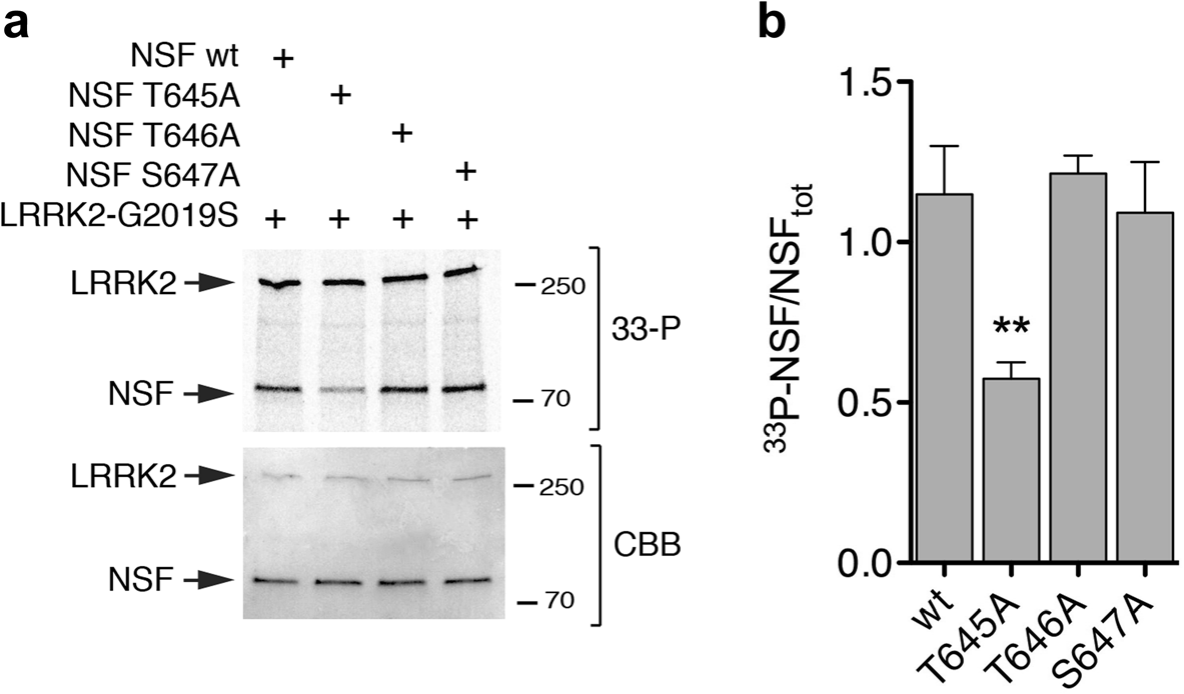
LRRK2 phosphorylates NSF at T645. **a** In vitro kinase assays with 3xFlag-LRRK2 G2019S and NSF wild-type or non-phosphorylatable mutants T645A, T646A and S647A mutants at 1:10 ratio LRRK2:NSF. The G2019S hyperactive mutant was used to maximize ^33^P incorporation. **b** Quantification of ^33^P incorporated by NSF (autoradiography, *upper panel*) controlled for total NSF (NSF_TOT_, coomassie staining, *lower panel*) from *n* = 4 independent experiments (bars represent the mean ± SEM). One way-ANOVA with Bonferroni’s post-test (**p < 0.01)

We next set out to identify NSF phosphorylation site(s) targeted by LRRK2. To achieve this, we used phospho-peptide enrichment coupled with liquid chromatography/tandem mass spectrometry (LC-MS/MS) analysis on purified NSF pretreated with alkaline phosphatase to eliminate possible cellular phosphorylation sites, and subsequently phosphorylated by LRRK2 in vitro. Under the experimental conditions used, we were able to achieve ≈ 80 % NSF sequence coverage (Additional file 1: Figure S4a) and identified the peptide ^639^KLLIIGTTSR^648^ as a *bona fide* phospho substrate. The MS analysis could not discriminate whether single/multiple phosphorylation occurred at T645, T646 or S647 or whether these sites may be multi-phosphorylated (Additional file 1: Figure S4b). This phosphopeptide was enriched following incubation with wild-type and G2019S LRRK2, but not in control samples (LRRK2 kinase dead or in the presence of 1 μM IN-1) indicating it contains specific LRRK2 phosphorylation site(s). We next validated the MS data by site-direct mutagenesis and in vitro kinase assays. Wild-type and phospho-deficient NSF mutants T645A, T646A and S647A were expressed and purified in HEK293T cells and subsequently incubated in vitro with catalytically active LRRK2 under phosphorylation permissive conditions. T645A displayed ~50 % reduction of ^33^P incorporation compared to NSF wild-type, T646A and S647A (**p < 0.01, One-Way ANOVA with Bonferroni’s post-test) (Fig. 4). Since NSF is also phosphorylated by PKC but within a different residue (S237) [26], we next assessed whether PKC is able to phosphorylate NSF at T645 to rule out any promiscuous effect. As shown in Additional file 1: Figure S5, we confirm that PKC efficiently phosphorylates NSF in vitro, but NSF^T645A^ exhibits similar ^33^P incorporation as NSF wild-type, suggesting that T645 is a LRRK2 specific phospho-site. Overall, our data indicate that T645 is a LRRK2 phosphorylation site within NSF.

### LRRK2-mediated phosphorylation increases NSF ATPase activity

**Fig. 5:**
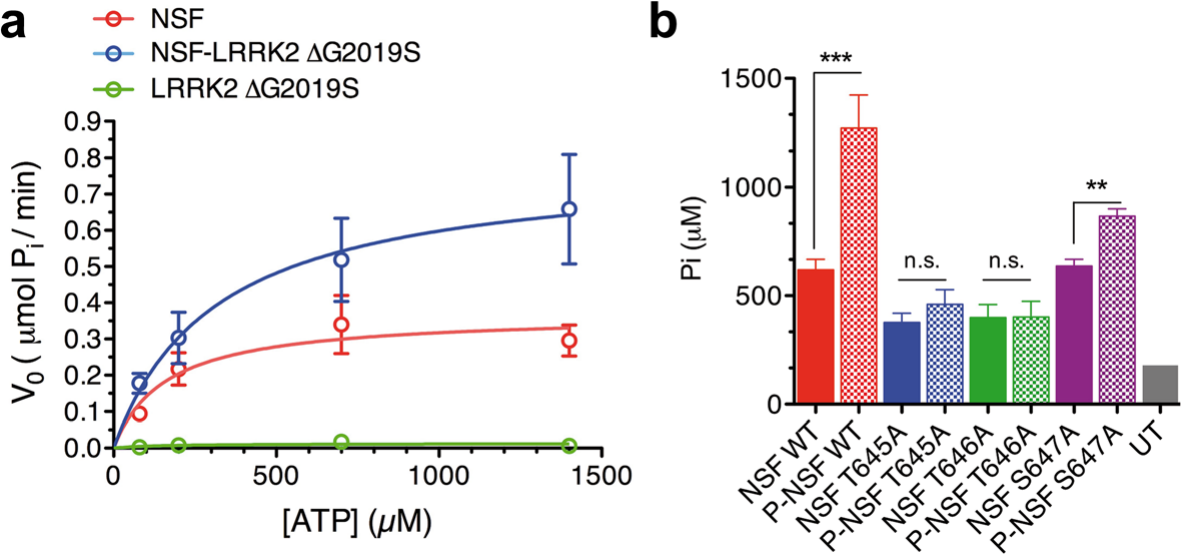
Phosphorylated NSF exhibits enhanced ATPase activity. **a** NSF ATPase activity was assessed with a Malachite green assays at 36nM NSF and increasing concentrations of ATP substrate (up to 1.4 mM) in the presence of NSF alone, NSF pre-phosphorylated by LRRK2-G2019S^970–2527^ (ΔG2019S) or LRRK2-G2019S^970–2527^ (ΔG2019S) alone (NSF:LRRK2 20:1). Data were fitted with the Michaelis-Menten kinetic model to determine kinetic constants. **b** Phosphate generated by ATP hydrolysis in the presence of NSF wild-type, NSF^T645A^ NSF^T646A^ NSF^S647A^ pre-phosphorylated or not by ΔG2019S was measured with the Malachite Green Assay at 120 min with an initial concentration of ATP of 1.4 mM (NSF^WT^ vs NSF^T645A^ **p < 0.01; NSF^WT^ vs P-NSF^WT^ ***p < 0.001; NSF^T645A^ vs P-NSF^T645A^ *p* > 0.05, non significant, n.s.; NSF^T646A^ vs P-NSF^T646A^ *p* > 0.05, n.s.; NSF^S647A^ vs P-NSF^S647A^ **p < 0.01; one-way ANOVA, Bonferroni’s post-test, *n* ≥ 3). UT = untrasfected cells subjected to Flag-affinity purification to monitor background activity (*n* = 1; excluded from the statistical analysis)

We next investigated whether LRRK2 mediated phosphorylation of NSF has a functional consequence on its ATPase activity. We first identified the optimal detergent concentrations at which both LRRK2 and NSF display maximal catalytic activity (0.007 % of polysorbate 20, the critical micelle concentration of the detergent; Additional file 1: Figure S6). Subsequently, full-length NSF was exposed to LRRK2-G2019S^970–2527^ or buffer in kinase assays conditions (with 50 μM ATP to minimize interference with the subsequent ATPase assay) for 30 min. As shown in Fig. 5a, NSF phosphorylated by LRRK2 exhibits increased ATPase activity (*K*_m_ = 355 ± 50 μM; *k*_cat_ = 40 ± 11 min^−1^; V_max_ = 0.95 ± 0.22 μmol/min, from *n* = 4 independent purifications) compared to unphosphorylated NSF (*K*_m_ = 178 ± 12 μM; *k*_cat_ = 19 ± 4 min^−1^; V_max_ = 0.37 ± 0.07 μmol/min, from *n* = 4 independent purifications). Given that we identified threonine 645 as a *bona fide* LRRK2 target, we next assessed the ATPase activity of NSF^T645A^, along with NSF wild-type, NSF^T646A^ and NSF^S647A^, pre-treated with LRRK2-G2019S^970–2527^ or buffer control in kinase assay conditions. As shown in Fig. 5b, NSF^T645A^ displays impaired ability to hydrolyze ATP and, importantly, activity could not be restored when NSF^T645A^ is pre-phosphorylated by LRRK2. Interestingly, the neighboring T646 mutated to alanine also exhibits impaired ATPase activity that cannot be recovered by LRRK2 phosphorylation, whereas NSF^S647A^, two residues apart from T645, displays ATPase activity similar to wild-type, and this activity is enhanced by LRRK2 phosphorylation. These results strongly indicate that T645 and T646 are critical for NSF catalytic activity. To rule out that NSF^T645A^ impaired activity was due to partial unfolding, we used circular dichroism (CD) and fluorescence spectroscopy to compare the secondary structures of NSF wild-type and NSF^T645A^. Tryptophan fluorescence is similar among wild-type and NSF^T645A^ (Additional file 1: Figure S7a). CD spectra also confirm that the overall folding is maintained (Additional file 1: Figure S7b). In addition, TEM imaging confirms that NSF^T645A^ retains the ability to form hexamers (Additional file 1: Figure S7c). Taken together, these data indicate that NSF phosphorylated by LRRK2 possesses enhanced ATPase activity and T645 is a crucial site for enzymatic catalysis.

### LRRK2-mediated phosphorylation of NSF increases the rate of SNARE complex disassembling

**Fig. 6:**
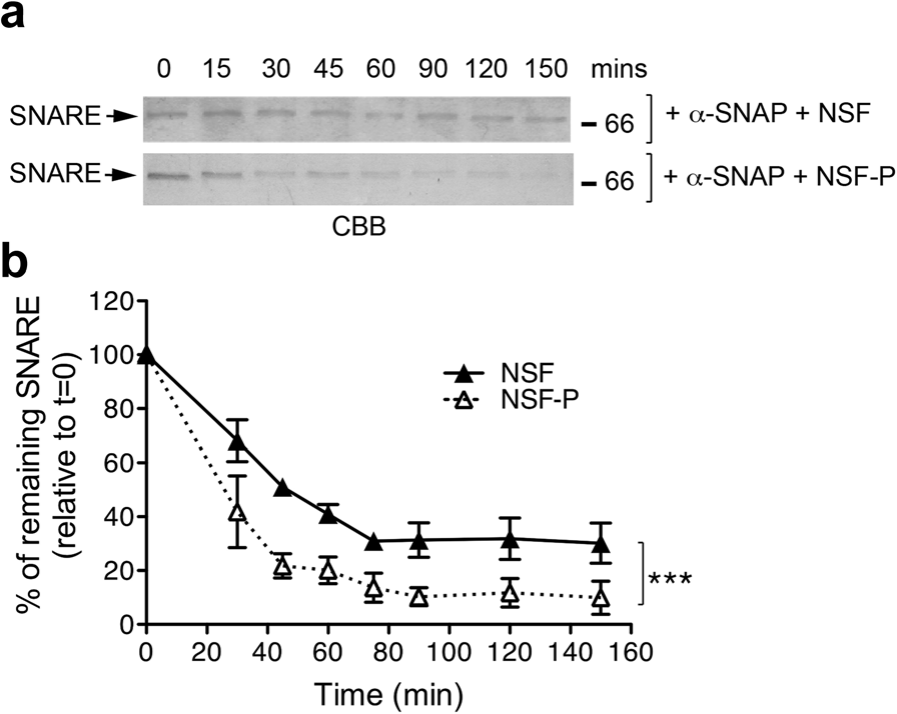
Phosphorylated NSF exhibits increased rate of SNARE complex disassembling in vitro. **a** Representative coomassie gels (CBB, coomassie brilliant blue) of SNARE complex incubated for increasing time with NSF phosphorylated or not by LRRK2-G2019S^970–2527^ in the presence of the co-factor alpha-SNAP. **b** Quantification of *n* = 3 independent experiments. Time points are relative to t = 0 which was set at 100 % (two-way ANOVA with Bonferroni’s post-test, ***p < 0.001)

NSF-mediated ATP hydrolysis promotes disassembly of the SNARE complex [33]. Given that NSF phosphorylation by LRRK2 increases its ATPase activity (Fig. 5), we next postulated that this also impacts upon the rate of SNARE complex dissociation. To test this, we monitored the kinetic of SNARE complex disassembling in vitro as previously described [34, 35]. We co-expressed in *E. coli* recombinant soluble syntaxin, SNAP-25 and 6xHis-VAMP and purified the assembled complex by IMAC affinity purification followed by size exclusion chromatography [35]. As shown in Additional file 1: Figure S8, the complex elutes as single band corresponding to the expected molecular weight for the soluble SNARE complex (68 kDa), which is dissociated into the three SNARE components upon heating. To assess whether LRRK2 phosphorylation on NSF impacts the rate of SNARE complex disassembling in vitro, we incubated SNARE complex (480 nM) with 1.5 μM of alpha-SNAP, an essential co-factor, and 24 nM of NSF (phosphorylated or not by LRRK2) in the presence of 2 mM of ATP, and subsequently analyzed the kinetic of SNARE complex disappearance over 150 min. Under these assay conditions, NSF phosphorylated by LRRK2 displayed a markedly increased efficiency in disassembling SNARE complex compared to non-phosphorylated NSF (Fig. 6a–b). These data further support the notion that LRRK2-mediated phosphorylation increases NSF catalytic activity with consequent acceleration of the disassembly of SNARE complexes in vitro.

## Discussion

Identification of heterologous substrates of LRRK2 kinase activity is essential for understanding the cellular pathways deregulated in PD caused by mutations in this gene [3]. Here we provided evidence that the presynaptic ATPase NSF is a substrate of LRRK2 kinase activity, and that phosphorylated NSF displays enhanced ATP hydrolysis and SNARE complex dissociation activity in vitro.

Multiple lines of evidence support a role of LRRK2 at the presynaptic compartment. We previously found that LRRK2 controls SV storage and mobilization within the recycling pool [20] and that this process is dependent on LRRK2 kinase activity [22]. Synaptosomes treated with LRRK2 inhibitors exhibit decreased evoked glutamate release [22] whereas elevated glutamate release and synaptic transmission were observed in LRRK2 G2019S knock-in mice [16]. Altogether, these data indicate that LRRK2 kinase may play an important role in modulating a step of the exo-endocytic pathway. LRRK2 is a complex kinase with several protein interaction domains, which was shown to assemble multiprotein complexes during signal transduction [11]. At the presynapse, LRRK2 interacts with several proteins [21]. Moreover, accumulating literature suggests that LRRK2 regulates SV dynamics *via* phosphorylation of presynaptic proteins, such as Snapin, and EndophilinA [36–38]. The present work provides evidence that NSF is not only an interactor but also a substrate of LRRK2 in vitro. Recently, another LRRK2 interactor, the ribosomal protein s15, has been shown to serve as LRRK2 substrate [39], further emphasizing the value of examining LRRK2 interacting proteins as potential kinase substrates.

Here, we demonstrated that LRRK2 phosphorylates NSF at T645. The functional consequence of this is that NSF hydrolyses ATP faster when phosphorylated by LRRK2 at this residue. The physiological relevance of this finding is further supported by the observed increased rate of SNARE complex disassembling in the presence of phosphorylated NSF. Indeed, we found T645A is characterized by a reduced endogenous ATPase activity. Given that T645 is located within the D2 domain of NSF, which is thought to be important for NSF oligomerization *via* ATP binding [40], these results are consistent with our current understanding of NSF function. Specifically, T645 is part of the beta strand S4 (aa 639–646), which stabilizes the hexamer through interaction with the neighboring alpha-helix H5 [41]. Therefore, T645 is predicted to be a key residue for protein oligomerization, which impacts the ability of the D1 domain to hydrolyze ATP. Clearly, further investigation is merited to determine whether LRRK2 phosphorylation at T645 directly alters NSF oligomerization.

Translating these findings into the neuronal context, these data imply that LRRK2 may play a role in tuning the kinetics of SV fusion by accelerating SNARE complex dissociation *via* NSF phosphorylation. Previous studies reported NSF as substrate of other serine-threonine kinases: NSF is phosphorylated by Pctaire1 at S569 in the D2 domain, and this phosphorylation reduces NSF oligomerization [25]; NSF is also phosphorylated by PKC in vitro at Ser-237 of the catalytic D1 domain which negatively regulate NSF binding to alpha-SNAP-SNARE complexes (Additional file 1: Figure S5) [26]. Therefore, both Pctaire1 and PKC appear to switch off NSF activity. In our current study, we report multiple strands of evidence that suggest that NSF phosphorylated by LRRK2 is more active in vitro, however it is still unknown whether this is replicated in vivo. Wild-type LRRK2 is characterized by a low basal activity that may become pathologically relevant in the presence of gain of function mutations. We predict that in the presence of pathological hyperactive LRRK2 variant, SV endocytosis may be abnormally fast. Such alteration could result in 1) increased neurotransmitter release or 2) impaired neurotransmitter release by accelerating SV endocytosis. While the second hypothesis would fit with the reduced dopamine release observed in mice expressing LRRK2 G2019S selectively in midbrain dopaminergic neurons [42], increased glutamate release has been reported in G2019S knock-in neurons [16] – consistent with the first hypothesis. Thus, additional research is needed to clearly identify the best representative model of LRRK2 function and dysfunction in the neuron.

We also provide robust evidence that the kinase activity of LRRK2 affects SV dynamics using two complementary models: LRRK2 inhibition and hyperactive LRRK2 (BAC hG2019S) in primary cortical neurons. The strong impairment of SV exo-endocytosis observed in the presence of pharmacological inhibition (Fig. 1, [22]) suggests that a consequence of therapeutic LRRK2 kinase inhibition might be alterations in the biology of the presynaptic compartment, likely impairing neurotransmitter release and synaptic function. These observations, together with the reported side effects in peripheral organs [43] suggest that additional strategies should be considered to target pathological LRRK2 function.

## Conclusions

In the present study, we report LRRK2 kinase as a positive regulator of NSF activity and SNARE complex disassembling in vitro. Future studies should also be directed at understanding whether this phosphorylation is relevant in the pathogenesis of PD.

## Methods

### Animals, neuron cultures and drugs

Housing and handling of mice were carried out in compliance with the guidelines established by the European Community Council (Directive 2010/63/EU of March 4th, 2014) and approved by the Italian Ministry of Health (IACUC 625). Non-transgenic wild-type and LRRK2 BAC hG2019S mice, back-crossed on a C57BL/6J strain, were obtained from Mayo Clinic (Jacksonville, FL, USA) through a collaboration with Dr. Heather Melrose [28]. Animals were kept following guidelines of Ministry of Education, Universities and Research (MIUR). Neuron cultures were prepared from either mouse cortexes or hippocampi obtained from embryonic day 15.5–16.5 mice (C57BL/6 J). High-density (750–1000 cells/mm^2^) and medium-density (150–200 cells/mm^2^) neuron cultures were plated and grown as described on 12-well plastic tissue culture plates (Iwaki; Bibby Sterilin Staffordshire, UK) or on 12 mm diameter coverslips put into 24-well plastic tissue culture plates (Iwaki) [44]. GSK-2578215A compound (Tocris Bioscience, Bristol, UK) or DMSO were added to culture media at the concentrations indicated through the text. For immunocytochemistry, primary cultured neurons were fixed with 4 % paraformaldehyde and probed with primary rabbit anti-NSF (1:200, D31C7, Cell Signaling, Danvers, MA, USA) and mouse anti-LRRK2 (1:200 N231B/34, NeuroMab, Davis, CA, USA) and secondary anti-mouse Alexa Fluor 488 and anti-rabbit Alexa Fluor 568 (Thermo Fisher, Waltham, MA USA).

### Plasmids and constructs

pCHMWS 3xFlag-tagged LRRK2 wild-type, K1906M and G2019S, 2x-Myc LRRK2 constructs have been previously described [31]. NSF constructs (full-length and domains) were cloned into p3XFLAG-CMV-7.1 vector (Sigma-Aldrich, St. Louis, MO, USA). NSF domains were amplified using forward primers with NotI overhang and reverse primers with KpnI overhang as following:

- N-Domain (1–205): forward 5′-AAGCTTGCGGCCGCCTTCGCGGGCCGGAGC-3′ and reverse 5′-TCGACTGGTACCTTAGCGATTTTCCTTGGTTTT-3′
- D1 domain (206-477aa): forward 5′-AAGCTTGCGGCCGCCCAATCAATTATCAATC-3′ and reverse 5′-TCGACTGGTACCTTATCTCGTCACTTGCAGGC-3′
- D2 domain (478-744aa): forward 5′-AAGCTTGCGGCCGCCGGAGACTTCCTTGCTTC-3′ and reverse 5′-TCGACTGGTACCTCAATCAAAATCAAGGGG-3′.

NSF mutants were generated using the QuickChange mutagenesis kit (Agilent Technologies, CA, USA) according to the manufacturer’s instructions. All plasmids were validated by restriction analysis and DNA sequencing.

### Cell culture and transfection

Human embryonic kidney cells (HEK293T) were cultured in Dulbecco’s modified Eagle’s medium (DMEM, Thermo Fisher, Waltham, MA USA) supplemented with 10 % fetal bovine serum (FBS, Thermo Fisher, Waltham, MA USA) at 37 °C and 5 % CO_2_. HEK293T were transiently transfected using linear polyethylenimine (PEI, Polysciences) with ratio DNA:PEI 1:2. 40 μg of DNA were dissolved in 1 ml of OPTI-MEM (Thermo Fisher, Waltham, MA USA) and 80 μl of PEI (40 μM) were added to 1 ml of OPTI-MEM. After 5 min of incubation the two solutions were mixed together and incubated for 20 min to allow the formation of DNA/PEI complexes. Then, the mix was added directly to the cells in Petri dishes of 15 cm^2^ and used after 48–72 h.

### Antibodies, SDS-PAGE and western blot analysis

Antibodies used for western blotting were as follows: anti-Flag M2 (1:10000, Sigma-Aldrich, St. Louis, MO, USA); anti-NSF (1:500, Cell Signaling, Danvers, MA, USA); anti-LRRK2 (1:1000, C41-2, Abcam, Cambridge, UK); anti-Synaptobrevin, anti-synaptophysin and anti-Synaptotagmin 1 (1:1000, Synaptic System, Göttingen, Germany).

Between 10 and 20 μg of protein samples were dissolved in 4–20 % Tris-glycine polyacrylamide gels (Biorad) in SDS/Tris-glycine running buffer. Precision Plus molecular weight markers (Biorad) were used for size estimation. Solubilized proteins were then transferred to polyvinylidenedifluoride (PVDF) membranes in transfer buffer containing 10 % methanol. The PVDF sheets were blocked in Tris-buffered saline plus 0.1 % Triton (TBS-T) plus 5 % nonfat dry milk for 1 h at 4 °C and then incubated overnight at 4 °C with primary antibody in TBS-T plus 5 % non-fat dry milk. The PVDF membranes were washed in TBS-T (3 × 10 min) at room temperature (RT) followed by incubation for 1 h at RT with horseradish peroxidase-conjugated anti-mouse IgG. Blots were then washed in TBS-T (4 × 10 min) at RT and rinsed in TBS, and immunoreactive proteins were visualized using enhanced chemiluminescence plus (ECL+, GE Healthcare, Waukesha, WI, USA). Densitometric analysis was carried out using Image J software.

### Protein purification

Human NSF with a N-terminal Flag tag or NSF domains were purified from HEK293T cells after transient transfection as described above. Cells were resuspended in 1 ml of a lysis buffer (20 mM Tris–HCl pH 7.5, 150 mM NaCl, 1 mM EDTA, 2.5 mM Na_4_P_2_O_7_, 1 mM beta-glycerophosphate, 1 mM Na_3_VO_4_, Protease Inhibitor Mixture (Sigma-Aldrich, St. Louis, MO, USA)) and then lysed with 5 cycles of freezing and thawing in liquid nitrogen. The cell lysate was collected after centrifugation at 18000xg for 40 min at 4 °C. The supernatant was incubate overnight with 40 μl of Anti-Flag M2 Affinity gel (Sigma-Aldrich, St. Louis, MO, USA) at 4 °C. After centrifugation, the supernatant was discarded and the beads with human NSF were washed with 1 ml of different buffers: WB1 (20 mM Tris–HCl pH 7.5, 500 mM NaCl) twice, WB2 (20 mM Tris–HCl pH 7.5, 350 mM NaCl) twice, WB3 (20 mM Tris–HCl pH 7.5, 150 mM NaCl) six times. The protein was then eluted by incubating the beads with 200 μl of 20 mM Tris–HCl pH 7.5, 150 mM NaCl or directly in the kinase assay buffer (25 mM Tris–HCl pH 7.5, 5 mM beta-glycerophosphate, 2 mM DTT, 0,1 mM Na_3_VO_4_, 10 mM MgCl_2_) with 150 ng/μl 3xFlag peptide and mixing the sample for about 2 h. The sample was centrifuged to pellet the resin and the supernatant was collected. Note that all the purification steps were carried out in the absence of detergent, a condition that resulted essential to maintain NSF folding and to detect specific phosphorylation by LRRK2. Purified human NSF was separated on SDS-PAGE and quantified by comparison with different concentrations of BSA (Bovine Serum Albumin). Proteins were electrophoretically resolved on 4–20 % Tris-glycine polyacrylamide gels (Biorad) using SDS/Tris-glycine running buffer. To estimate the molecular weight of proteins Precision Plus molecular weight marker (Biorad) was used. After the run, proteins were stained with Coomassie Brillant blue to enable the quantification with ImageJ software.

### Synaptosomes preparation and immunoprecipitation

Brains from adult rats were quickly removed and the cerebral cortex dissected out at 4 °C. Purified synaptosomes were prepared on Percoll gradients (Sigma-Aldrich, St Louis, MO, USA) essentially according to Nakamura et al. with minor modifications [45]. Briefly, the tissue was homogenized in 14 volumes of 0.32 M sucrose, Tris–HCl pH 7.4, using a glass-teflon tissue grinder (clearance 0.25 mm, 12 up–down strokes in about 1 min). The homogenate was centrifuged (5 min, 1000 g at 4 °C) to remove nuclei and debris and the supernatant was gently stratified on a discontinuous Percoll gradient (2, 6, 10, and 20 % v/v in Tris-buffered sucrose) and centrifuged at 33,500 g for 5 min at 4 °C.

The layer between 10 and 20 % Percoll (synaptosomal fraction) was collected, washed by centrifugation and resuspended in RIPA buffer (NaCl 150 mM, Tris 50 mM (pH 7.4), NP40 (1%v/v), SDS (0.1%v/v) and protease inhibitors). To precipitate the immunocomplexes the extract was incubated for 2 h at RT with anti-LRRK2 antibodies (10 μg/sample; MJFF C41-2, Abcam, Cambridge, UK) or a control rabbit IgG (10 μg/sample; Sigma-Aldrich, St. Louis, MO, USA) conjugated with 25 μl of settled prewashed protein G-Sepharose beads (GE-Healthcare, Waukesha, WI, USA). The eluted proteins were separated by SDS-PAGE, transferred onto nitrocellulose membrane (GE-Healthcare, Waukesha, WI, USA) and analyzed by western-blotting with anti-LRRK2 and anti-NSF (Cell Signaling, Danvers, MA, USA) antibodies. Western-blotting with anti-synaptotagmin 1, anti-synaptophysin and anti-synaptobrevin were performed to confirm purity of synaptosomal preparation.

### Pull-down assays

NSF domains and full length NSF were purified after transient transfection from HEK293T cells. Cells were harvested in 500 μl of Lysis buffer (50 mM Tris–HCl pH 7.5, 1 mM EDTA, 2.5 mM Na_4_P_2_O_7_, 1 mM beta-glycerophosphate, 1 mM Na_3_VO_4_, 0.27 M Sucrose, 1 % Triton X-100, Protease Inhibitor Mixture (Sigma-Aldrich, St. Louis, MO, USA)). The cell lysate was then centrifuged at 18000xg for 30 min at 4 °C. Subsequently, the lysate was incubated overnight with 20 μl of Anti-Flag M2 Affinity gel (Sigma-Aldrich, St. Louis, MO, USA) at 4 °C. After centrifugation, the supernatant was discarded and the beads with NSF proteins were washed three times with 1 ml of a Washing buffer (50 mM Tris–HCl pH 7.5, 1 mM EDTA, 0.27 M Sucrose, 250 mM NaCl, 0.02 % Triton X-100) and resuspended in 100 μl of the same buffer. Proteins were loaded on an SDS-PAGE gel and their concentration was quantified measuring the intensity of the band against known BSA standards with ImageJ software.

Proteins were subsequently adjusted to the same concentration (2 μM) and incubated with 600 μl mouse brain lysate (2.5 mg/ml concentrated) overnight at 4 °C. The day after, resins were boiled with sample buffer, loaded into a SDS-PAGE gel and transferred onto PVDF membranes.

### Size Exclusion Chromatography (SEC) and dot blot analysis

Flag-NSF alone or Flag-NSF and 2xmyc-LRRK2 transfected HEK293T cells were lysed in 500 μl of lysis buffer containing 0.06 % (v/v) Triton X-100 and centrifuged. Cell lysates clarified were separated on a Superose 6 10/300 column (Ge Healthcare, Waukesha, WI, USA) pre-equilibrated with 20 mM Tris–HCl pH 7.5, 150 mM NaCl and 0.06 % (v/v) Triton X-100. The flow rate used was 0.5 ml/min. A calibration curve was produced using the following proteins and relative elution volumes: 7.5 ml for Blue Dextran (void volume), 11.5 ml for hemocyanin from *Carcinus aestuarii* (900 kDa), 12 ml for thyroglobulin (669 kDa), 14 ml for ferritin (440 kDa) and 12.5 ml for catalase (232 kDa). Fractions of 0.25 ml were collected and spotted onto a nitrocellulose membrane and analyzed by dot blot. The membrane was blocked with 10 % milk in TTBS and incubated with mouse monoclonal anti-Flag M2-peroxidase (Sigma-Aldrich, St. Louis, MO, USA) or anti-myc (Roche) in TTBS with 10 % milk. A secondary rabbit antibody (Sigma-Aldrich, St. Louis, MO, USA) was used to stain the anti-myc. Immunoproteins were visualized using ECL (GE, Healthcare, Waukesha, WI, USA).

### Electron microscopy

Purified NSF proteins were incubated with 1 mM ATP and 2 mM MgCl_2_. A total of 15 ng of protein was adsorbed few minutes to a glow-discharged carbon-coated copper grid, washed with deionized water, and stained with 1 % uranyl acetate. Images were collected using a Fei Tecnai T12 electron microscope equipped with a LaB6 filament and operated at an acceleration voltage of 100 kV.

### In vitro kinase assay

Purified NSFs eluted in kinase assay buffer were incubated with LRRK2 proteins dissolved in kinase buffer for 1 h at 30 °C in the presence of ^33^P-ATP (1 μCi) and 10 μM cold ATP as previously described [31].

Incorporated ^33^P-ATP was detected by autoradiography or by Phospho-Imager system (Cyclone, Perkin-Elmer). The same membranes were probed with anti-Flag antibody for total protein loading and analyzed using ImageJ software.

### SypHy assay

We infected DIV4 primary neurons with viruses expressing sypHy, a fusion construct of synaptophysin and super ecliptic pHluorin [29]. At DIV14 neurons were treated with DMSO (control) or GSK2578215A (0.2 μM, 2 h). Syphy positive boutons were assayed in a stimulation chamber on the stage of a Zeiss Axiovert 200 M equipped with a mono-chromator (Poly V) and a cooled CCD camera (PCO, Imago QE), both from TILL photonics (Gräfelfing, Germany). The assay was carried out as described previously [46]. Briefly, cells were submerged in 500 μl of KRH buffer (125 mM NaCl, 5 mM KCl, 1.8 mM CaCl_2_ 2.6 mM MgSO_4_ 5 mM Hepes, pH 7.2) in presence of APV (2 μM, Sigma-Aldrich, St. Louis, MO, USA) and CNQX (2 μM, Sigma-Aldrich, St. Louis, MO, USA). SypHy was excited at 475 nm and its fluorescence emission collected at 525 nm using a 60X, 1.1 NA water immersion objective. Images were acquired every second for 200 s using TillVision software (TILL Photonics). At frame 30, cells were stimulated with 40 action potential (AP, 20Hz) then at frame 70 with 300 AP (20 Hz). Total fluorescence was measured upon incubation with 50 mM NH_4_Cl. Quantitative measurements of the fluorescence intensity at individual boutons were obtained by averaging a selected area of pixel intensities using ImageJ. Net fluorescence changes (ΔF) were obtained by subtracting the average intensity of the first 15 frames (F0) from the intensity of each frame (Ft) for individual boutons and normalized F0 (ΔF/F0). The fluorescence increase and decay, reflect exo- and endocytosis, respectively [29]. Both the fluorescence upstroke and decay were fitted with a single exponential τ (τ_upstroke_ and τ_decay_ respectively). Data are expressed as mean ± SEM and statistical significance was assessed by unpaired two-tailed Student’s *t* test (GraphPad Prism).

### Exo/endocytotic assay

The endocytosis assay to monitor SV recycling was performed using rabbit polyclonal antibodies directed against the intravesicular domain of synaptotagmin1 (Synaptic System), applied for 5 min at RT on the cultures, as described previously [30]. Incubations with the antibody (1:400) were performed in Tyrode solution containing 124 mM NaCl, 5 mM KCl, 2 mM MgCl_2_, 30 mM glucose, 25 mM HEPES, pH 7.4 and 2 mM CaCl_2_. After fixation and permeabilization, a synaptophysin counter staining with mouse anti synaptophysin, 1:400 (Sigma-Aldrich) visualized the totality of synaptic vesicles. Acquired images were processed and quantitatively analyzed with ImageJ software as previously described [47]. Briefly, cultures were infected at DIV4 with GPF expressing viruses and assayed at DIV14 as in [22]. GFP positive processes were manually tracked and the number of synaptotagmin and synaptophysin positive clusters and synaptophysin positive clusters present in the region of interest were automatically counted.

### Proteins digestion

Approximately 2 μg of purified NSF pre-dephosphorylated with alkaline phosphatase (Promega) and subsequently phosphorylated or not with LRRK2 in the presence of 100 μM ATP were loaded into a SDS-precasted gel (Biorad). Gel slices corresponding to purified NSF were excised, cut in smaller pieces, dehydrated with 100 μl of acetonitrile (ACN) for 10 min, then dried under vacuum. A protein reduction step was performed with 100 μl of freshly prepared 10 mM Dithiothreitol (DTT, Fluka) in 50 mM NH_4_HCO_3_, at 56 °C. After 1 h DTT solution was discarded and 100 μl of a freshly prepared solution of 55 mM iodoacetamide (Sigma-Aldrich, St. Louis, MO, USA) in 50 mM NH_4_HCO_3_ was added to the gel pieces for 45 min at room temperature and in the dark. Gel pieces were washed 4 times (10 min each) alternating 100 μl of 25 mM NH_4_HCO_3_ and 100 μl of ACN, dried under vacuum, and suspended in 20 μl of a sequencing grade modified trypsin solution (Promega, 12.5 ng/mL in 25 mM NH_4_HCO_3_). Digestion was performed overnight at 37 °C. Peptides were extracted with three changes (50 μl each) of 50 % ACN/0.1 % formic acid (FA, Fluka). Samples were dried under vacuum and stored at −20 °C till the phosphopeptide enrichment procedure was performed.

### Enrichment of phosphopeptides

Phosphopeptides were enriched with home made micro-columns of TiO_2_ as previously described [48]. TiO_2_ micro-columns were conditioned twice with 50 μl of ACN and twice with loading buffer (80 % ACN/6 % trifluoroacetic acid (TFA, Riedel-de Haën)). Samples were suspended in 50 μl of loading buffer and slowly loaded into the columns, which were then washed twice with 50 μl of loading buffer and twice with washing buffer (0.1 % TFA). Phosphopeptides bound to TiO_2_ were eluted with 50 μl of freshly prepared 5 % NH_4_OH and subsequently with 50 μl of 50 % ACN/0.1 % FA. Samples were immediately acidified by adding 5 μl of 100 % FA and dried under vacuum.

### Mass spectrometry analysis

Mass spectrometry analysis of phosphopeptides was performed with a LTQ-Orbitrap XL mass spectrometer (Thermo Fisher Scientific) coupled online with a nano-HPLC Ultimate 3000 (Dionex-Thermo Fisher Scientific). Samples were dissolved in 30 μl of 3 % ACN/0.1 % FA and for every analysis 8 μl of sample were loaded at a flow rate of 8 μl/min into a trap column (300 mm I.D., 300 Å, C18, 3 mm; SGE Analytical Science). Samples were injected into a home-made 10 cm pico-frit capillary column (75 μm I.D., 15 μm tip; New Objective) packed with C18 material (Aeris Peptide 3.6 um XB-C18, Phenomenex). Peptides were separated using a linear gradient from 3 to 40 % of ACN/0.1 FA in 20 min at a flow rate of 250 nl/min.

To increase the confidence in the identification of phosphopeptides, the MS analysis of each sample was performed with three different acquisition methods, as reported in [49]. A MS^2^ data dependent acquisition (1 full-MS scan in the range 300–1700 Da on the Orbitrap with a resolution of 60,000, followed by MS/MS spectra acquired in the linear ion trap for the ten most abundant ions); a MS^3^ neutral loss-triggered dependent acquisition (one full-MS scan on the Orbitrap, followed by MS/MS scans on the three most intense ions and by MS^3^ upon detection of neutral loss of phosphoric acid in MS^2^ spectra); a Multi Stage Acquisition (MSA) (1 full-MS scan at a resolution of 60,000 followed by MS/MS scans on the three most abundant ions with the activation of neutral loss product without an additional isolation cycle).

Raw data files were analyzed with Proteome Discoverer software (version 1.4, Thermo Fisher Scientific) connected to a Mascot Server version 2.2.4 (Matrix Science, UK) and a SequestHT search engine version 28.0 (Thermo Fisher Scientific) against the Uniprot Human Database (version 2013.11.13 used by SequestHT, version 2014.04.16 used by Mascot). Trypsin was set as digesting enzyme with up to two missed-cleavages. Carbamidomethyl cysteine was set as fixed modification, while phosphorylation of Ser/Thr/Tyr and methionine oxidation were set as variable modifications. Peptide and fragment tolerance were 10 ppm and 0.6 Da respectively. Percolator was used to calculate False Discovery Rate (FDR) based on the search against the corresponding randomized database. MS/MS spectra of phosphopeptides were manually inspected for confirmation and assignment of phosphorylation sites.

### ATPase enzymatic assay

NSF ATPase activity was quantified using the Malachite Green Assay by measuring the release of inorganic phosphate (Pi) due to the ATP hydrolysis with spectrophotometer. The assay was adapted from the method of Lanzetta et al. [50]. The Malachite Green Stock solution used for the assay was a mixture of two different solutions (one with 34 mg Malachite Green oxalate salt (Sigma-Aldrich. St. Louis, MO, USA) into 40 ml HCl 1 M and the other with 1 g (NH_4_)_2_MoO_4_ (Sigma-Aldrich, St. Louis, MO, USA) into 14 ml HCl 4 M to a final volume of 100 ml with distilled water and then filtered through 0,45 nm. The concentration of human NSF used for the ATPase assay was 216 nM (36 nM hexameric concentration) with different ATP concentration. Reaction was performed at 37 °C and followed for 120 min. The time point aliquots collected (20 μl) were mixed with 150 μl of Malachite Green stock solution until the solution became homogenous and the absorbance measured at 640 nm using a corresponding Malachite Green solution as blank. The values of absorbance were then converted into μmol of free Pi in solution using a standard curve. To reported values for the kinetic constants (*K*_m_, *k*_cat_ and V_max_) were obtained by data fitting with the Michaelis-Menten kinetic model (Y = Vmax*S/(Km + [S])).

### Reverse-phase HPLC ATPase assay

To determine the ATPase activity of NSF, 500 or 700 μM ATP was added to 0.2 μM 3xFlag-NSF wt. Proteins were purified as previously described and incubated at 37 °C for 1 h in the same kinase buffers and conditions of the Malachite Green Assay. At the reported time-points, aliquots (20 μl) were taken up to 120 min and heated for 3 min at 95 °C with 0.1 M of EDTA to stop the reaction. Samples were stored at −80 °C. Reverse Phase High-Performance Liquid Chromatography (RP-HPLC) was used to monitor the amount of ATP and ADP present in the sample. Nucleotides were separated on a Jupiter 5u C4 300A (Phenomenex) column using an Agilent HP 1100 HPLC, pre-equilibrated with 50 mM NaH_2_PO_4_ pH 6.5, 10 mM Tetra-n-butylammonium bromide and 4 % ACN. The flow-rate used was 0.5 ml/min and the amount of the nucleotides was monitored measuring the increase in area of the peak corresponding to ADP measured at 256 nm with a total run time of 35 min. To convert this value to the Pi released by the reaction, a standard curve generated with different ADP concentration was used. ADP concentrations detected in the assay were plotted as a function of time and an equation was obtained through linear regression with GraphPad Prism 5.

### Recombinant alpha SNAP and SNARE proteins production

Rat alpha-SNAP cloned in pET28 plasmid in fusion with a His-tag was a kind gift of Dr. Reinhard Jahn, Max-Planck-Institute, Göttingen). Alpha-SNAP was subsequently expressed in *E. Coli* in BL21(DE3) strain. Bacteria were grown at 37 °C to an OD at 600 nm of 0.4–0.6, then induced with 0.25 mM isopropyl β-D-1-thiogalactopyranoside (IPTG) for 4 h. Cells were then harvested by centrifugation and the pellet of 250 ml of culture was resuspended in 5–10 ml of Tris–HCl pH 8.0. Phenylmethylsulfonyl fluoride (PMSF) 100 μM and a cocktail of protease inhibitors were added to the cells 1:100 (v/v) that were subsequently subjected to one French Press cycles (Constant Systems Ltd). The cell homogenate was centrifuged and the supernatant loaded onto a Co^2+^ affinity column and eluted with a 0–500 mM linear gradient of imidazole at 0.5 ml/min. Protein solution was dialyzed versus Tris–HCl 20 mM pH 7.5, NaCl 150 mM.

Soluble SNARE complex was obtained by co-expression of wild type SNAP-25A, of syntaxin-1A and of His-tagged VAMP2(1–96) using the Duet expression system (Novagen) in *E. Coli* in BL21(DE3) strain. VAMP2(1–96)-His6-TEV pACYC-Duet and syntaxin-1A/SNAP-25A pET-Duet were a kind gift of Prof. A. Brunger (Stanford University, California) [34]. Bacteria were grown at 37 °C to an OD at 600 nm of 0.6–0.8, then induced with 0.5 mM IPTG for 4 h. Cell pellets of 250 ml of culture were suspended in 10 ml of 50 mM NaPi, pH 8.0, 300 mM NaCl, 20 mM imidazole and 0.5 mM Tris(2-carboxyethyl)phosphine (TCEP) (SNARE buffer), supplemented with PMSF 100 μM and protease inhibitors cocktail. Cell were lyses by two French Press cycles (Constant Systems Ltd) and the lysate was clarified by centrifugation for 1 h at 15,000 g at 4 °C. The supernatant was loaded onto a 1-ml Ni^2+^ affinity column, washed with 20 ml of SNARE buffer containing 7.5 M urea and then with 20 ml of SNARE buffer. The complex was then eluted with SNARE buffer containing 350 mM imidazole. After elution, SNARE complex was subjected to size exclusion chromatography using a Superdex 200 10/300 (GE Helthcare) that was equilibrated with 50 mM Tris–HCl, pH 7.5, 100 mM NaCl. The SNARE complex was checked and quantified by SDS-PAGE.

### SNARE dissociation assay

As previously described [34] the SNARE dissociation assays were performed at 37 °C in 240 μl in a 1.5 ml micro-tube. The assay buffer was composed of 25 mM Tris–HCl pH 7.5, 5 mM β–glycerophosphate, 2 mM dithiotreitol (DTT), 0.1 mM Na_3_VO_4_, 10 mM MgCl_2_ and 0.007 % polysorbate 20. Subsequently 1.5 μM αSNAP, 480 nM SNAREs, 24 nM NSF (hexameric concentration) phosphorylated or not by LRRK2 (ratio NSF:LRRK2 20:1) were added in the presence of 2 mM ATP to start the reaction. At defined time points, an aliquot (20 μl) was collected and loaded into an SDS-PAGE gel without boiling the samples: being the SNARE complex is SDS-resistant, it runs as a single band on SDS-PAGE gel. The intensity of each SNARE complex band was calculated and normalized to its time zero. The experiments were performed in triplicate up to 150 min of reaction.

### Circular Dichroism (CD)

CD measurements were carried out on a JASCO J-810 spectropolarimeter interfaced with a personal computer. The CD spectra were acquired and processed using the J-700 software for Windows. All experiments were done at room temperature using an optical path length of 0.2 cm. The wavelength range of the measurements was 197–250 nm, using a bandwidth of 2 nm and a time constant of 8 s at a scan speed of 50 nm/min. The signal to noise ratio was improved by accumulating four scans. Spectra were acquired using purified proteins in the elution buffer (20 mM Tris–HCl pH 7.5, 150 mM NaCl and 0.007 % polysorbate-20) using the same buffer with 3xFlag peptide as a control. All the spectra are reported in terms of mean residue molar ellipticity (deg cm^2^ dmol^−1^). Protein concentrations in the samples were determined by SDS-PAGE and all the spectra were normalized for the measured protein concentration.

### Intrinsic fluorescence

Fluorescence emission spectra were recorded on a Cary Eclipse fluorescence spectrophotometer (Varian, Agilent Technologies, Santa Clara, CA) using the Cary Eclipse program. Sample measurements were carried out using optical path length of 10 mm. Fluorescence spectra were obtained using an excitation wavelength of 288 nm, with an excitation bandwidth of 5 nm and slit width of 10 nm. Emission spectra were recorded between 300 and 400 nm at a scan rate of 30 nm/sec. Spectra were acquired using 80 nM proteins in 20 mM Tris/HCl buffer (pH 7.5), 150 mM NaCl and 0.02 % Tween 20.

### Statistical analysis

All quantitative data are expressed as mean ± SEM and represent at least three independent sets of experiments. Significance of differences between two groups was assessed by two-tailed unpaired *t*-test or one-way or two-way ANOVA with Bonferroni’s post-test when more than two groups were compared. Significance was set at *p* < 0.05.

## Additional file

Additional file 1:
**Figure S1.** Expression and phosphorylation of LRRK2 in primary cortical neurons subjected to SypHy assays. **Figure S2.** Biochemical validation of recombinant human NSF. **Figure S3.** LRRK2 phosphorylation of NSF occurs within the first 30 minutes. **Figure S4.** LC-MS/MS analysis of NSF phosphorylated by LRRK2. **Figure S5.** PKC does not phosphorylate NSF at T645. **Figure S6.** Evaluation of optimal detergent concentration for LRRK2 and NSF activities. **Figure S7.** NSF wild-type and T645A have similar overall folding and T645A forms hexamers. **Figure S8.** Expression and purification of SDS-resistant SNARE complex. (DOCX 2062 kb)

## Abbreviations

NSF: n-ethylmaleimide sensitive fusion
LRRK2: leucine-rich repeat kinase 2
SNARE: soluble NSF-attachment protein receptor
PD: Parkinson’s disease
ANOVA: analysis of variance
TBS: tris-buffered saline
SV: synaptic vesicles
sypHy: synaptophysin-pHluorin
CD: circular dichroism
MS: mass spectrometry
TEM: transmission electron microscopy
DIV: days in vitro
