# Supplementary material for: LRRK2 phosphorylates pre-synaptic N-ethylmaleimide sensitive fusion (NSF) protein enhancing its ATPase activity and SNARE complex disassembling rate
Source: Mol Neurodegener. 2016 Jan 13;11:1. doi: 10.1186/s13024-015-0066-z (PMC4711005; doi:10.1186/s13024-015-0066-z)
Supplement: Additional file 1: — Figure S1. Expression and phosphorylation of LRRK2 in primary cortical neurons subjected to SypHy assays. Figure S2. Biochemical validation of recombinant human NSF. Figure S3. LRRK2 phosphorylation of NSF occurs within the first 30 minutes. Figure S4. LC-MS/MS analysis of NSF phosphorylated by LRRK2. Figure S5. PKC does not phosphorylate NSF at T645. Figure S6. Evaluation of optimal detergent concentration for LRRK2 and NSF activities. Figure S7. NSF wild-type and T645A have similar overall folding and T645A forms hexamers. Figure S8. Expression and purification of SDS-resistant SNARE complex. (DOCX 2062 kb) [file 13024_2015_66_MOESM1_ESM.docx]

**Additional file 1**

**LRRK2 phosphorylates pre-synaptic N-ethylmaleimide sensitive fusion (NSF) protein enhancing its ATPase activity and SNARE complex disassembling rate**

Elisa Belluzzi^,^ Adriano Gonnelli, Maria-Daniela Cirnaru, Antonella Marte, Nicoletta Plotegher, Isabella Russo, Laura Civiero, Susanna Cogo, Maria Perèz Carrion, Cinzia Franchin, Giorgio Arrigoni, Mariano Beltramini, Luigi Bubacco, Franco Onofri, Giovanni Piccoli, Elisa Greggio


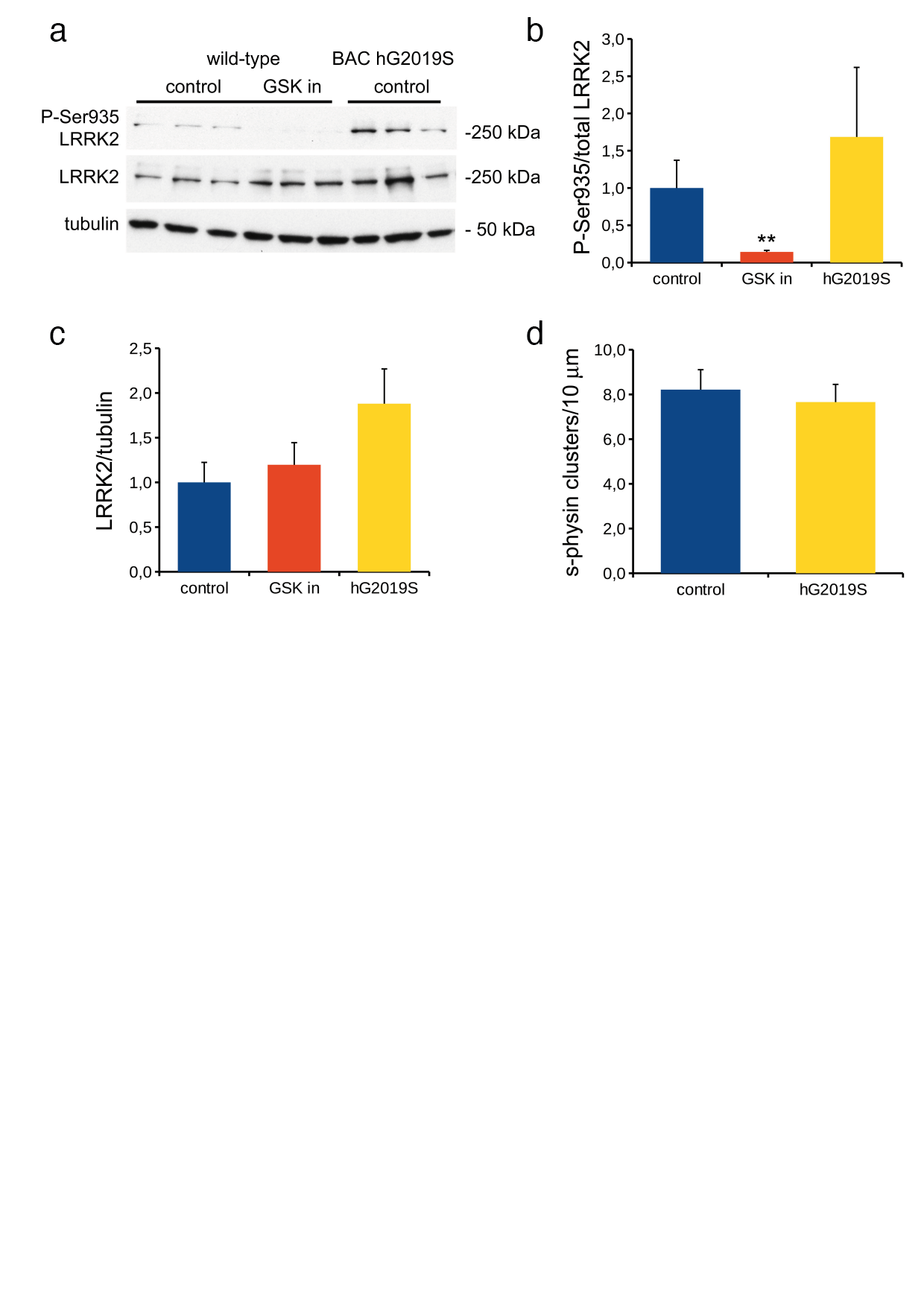


**Figure S1. Expression and phosphorylation of LRRK2 in primary cortical neurons subjected to SypHy assays.** (a) Cortical neurons from wild-type and BAC hG2019S mice were kept in culture until DIV14, treated or not with LRRK2 inhibitor GSK2578215A (GSK in, 0.2 μM, 2h) and assayed for western blotting. Samples were processed to measure LRRK2 phosphorylation at Ser935, total LRRK2 and tubulin level. Graphs report P-Ser935 level normalized on total LRRK2 level (b), total LRRK2 level normalized on tubulin amount (c). Data are expressed as mean ± SEM, n=3 (** p<0.01 versus control, ANOVA). (d) Total number of SV pools was not altered in BAC hG2019S neurons. The graph reports number of synaptophysin-positive clusters per 10 µm of GFP-positive process.


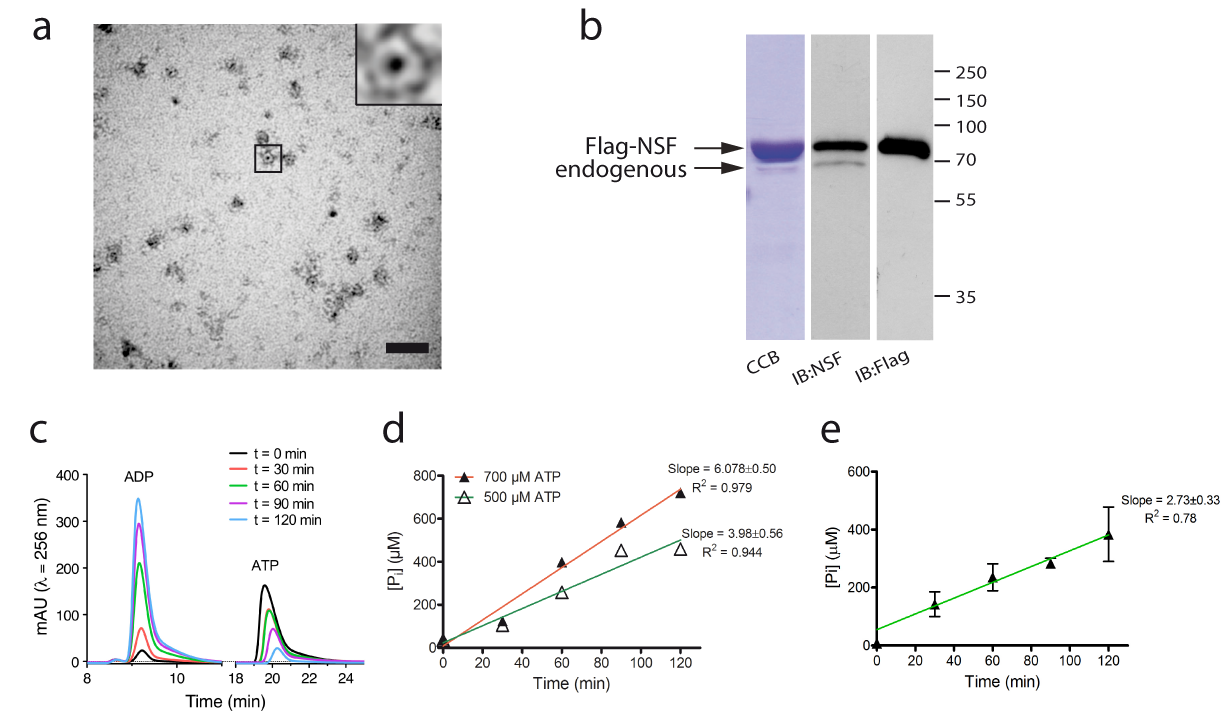


**Figure S2. Biochemical validation of recombinant human NSF.** (a) Representative TEM micrograph of purified Flag NSF wild type (20 ng/µl) with 1 mM ATP and 4 mM MgCl_2_ showed the typical hexameric structure (scale bar 50 nm). (b) Recombinant human NSF co-purifies with endogenous NSF as confirmed by western blot analysis with anti-flag and anti-NSF antibodies. (c) Isocratic RP-HPLC profile of the two different nucleotide peaks over time ([ATP] = 0.7 mM). The ATPase activity of NSF was evaluated also with the rate of inorganic phosphate (P_i_) released measuring the area under the peak of ADP with RP-HPLC (d) and with the Malachite Green Enzyme Assay ([ATP] = 0.5 mM) collecting aliquots as a function of time (n = 5) and subtracting a control purification of untransfected cells (e). Velocities of the reactions (slopes) were calculated by linear regression using GraphPad Prism 5 software.


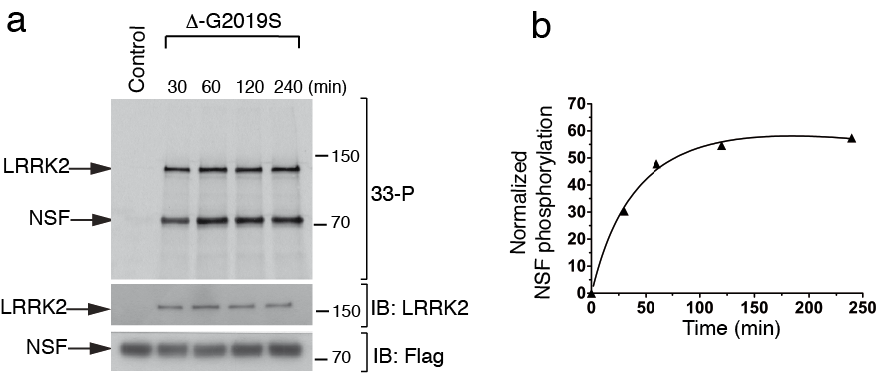


**Figure S3. LRRK2 phosphorylation of NSF occurs within the first 30 minutes.**

(a) In vitro kinase assays with LRRK2-G2019S^970-2527^ and NSF up to 240 minute reaction time. (b) Quantification of radioactivity incorporated by NSF over time.


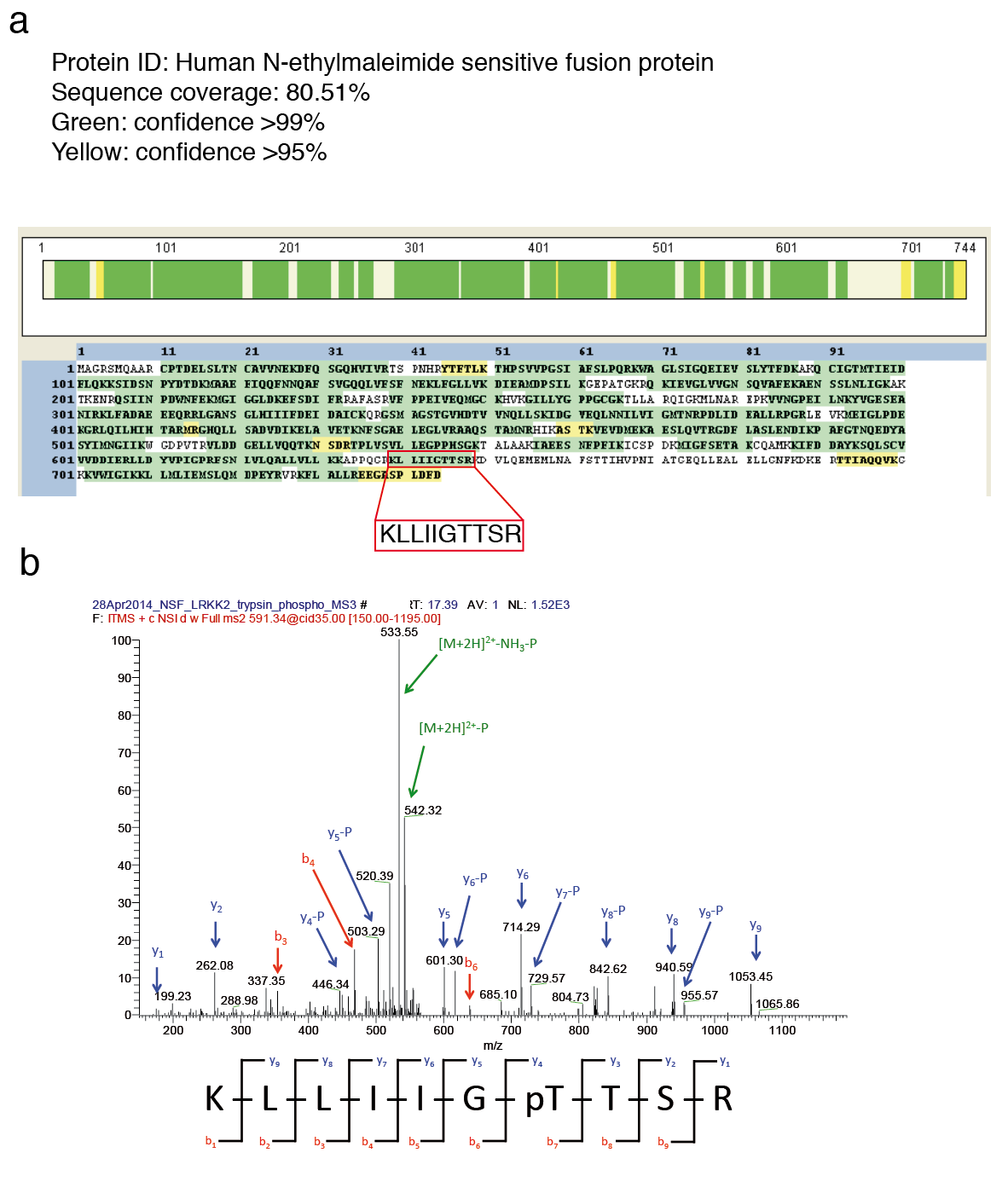


**Figure S4. LC-MS/MS analysis of NSF phosphorylated by LRRK2.**

(a) Sequence coverage of NSF phosphorylated by LRRK2. (b) Manually annotated MS/MS spectrum relative to the monophosphorylated peptide KLLIIGTTSR from NSF. Charge: +2, Monoisotopic m/z: 591.33636 Da (+0.03 mmu/+0.04 ppm respect to the theoretical m/z), MH+: 1181.66545 Da. Identified with Mascot with an IonScore of 60 (Exp Value of 2.5E-005). The presence of the fragment y2 suggests that the phosphorylation site is probably not located at the level of the serine residue. The fragmentation pattern is compatible with a phosphorylation at T645 or T646.


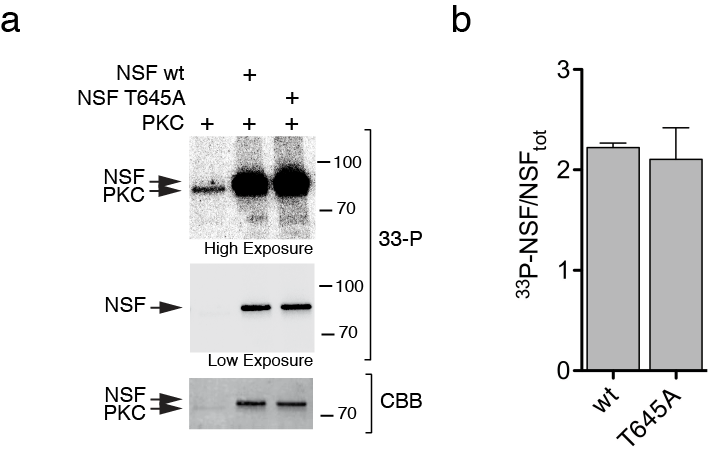


**Figure S5. PKC does not phosphorylate NSF at T645.**

(a) In vitro kinase assays with PKC and NSF (1:10, PKC:NSF). Upper panel shows a high exposure image where autophosphorylation of PKC can be observed. Middle panel represent a low exposure phosphoscreen. Lower panel shows a coomassie of the same membrane to normalize for protein loading. (b) Quantification of radioactivity incorporated by NSF wild-type and T645A (n=3 independent experiments).


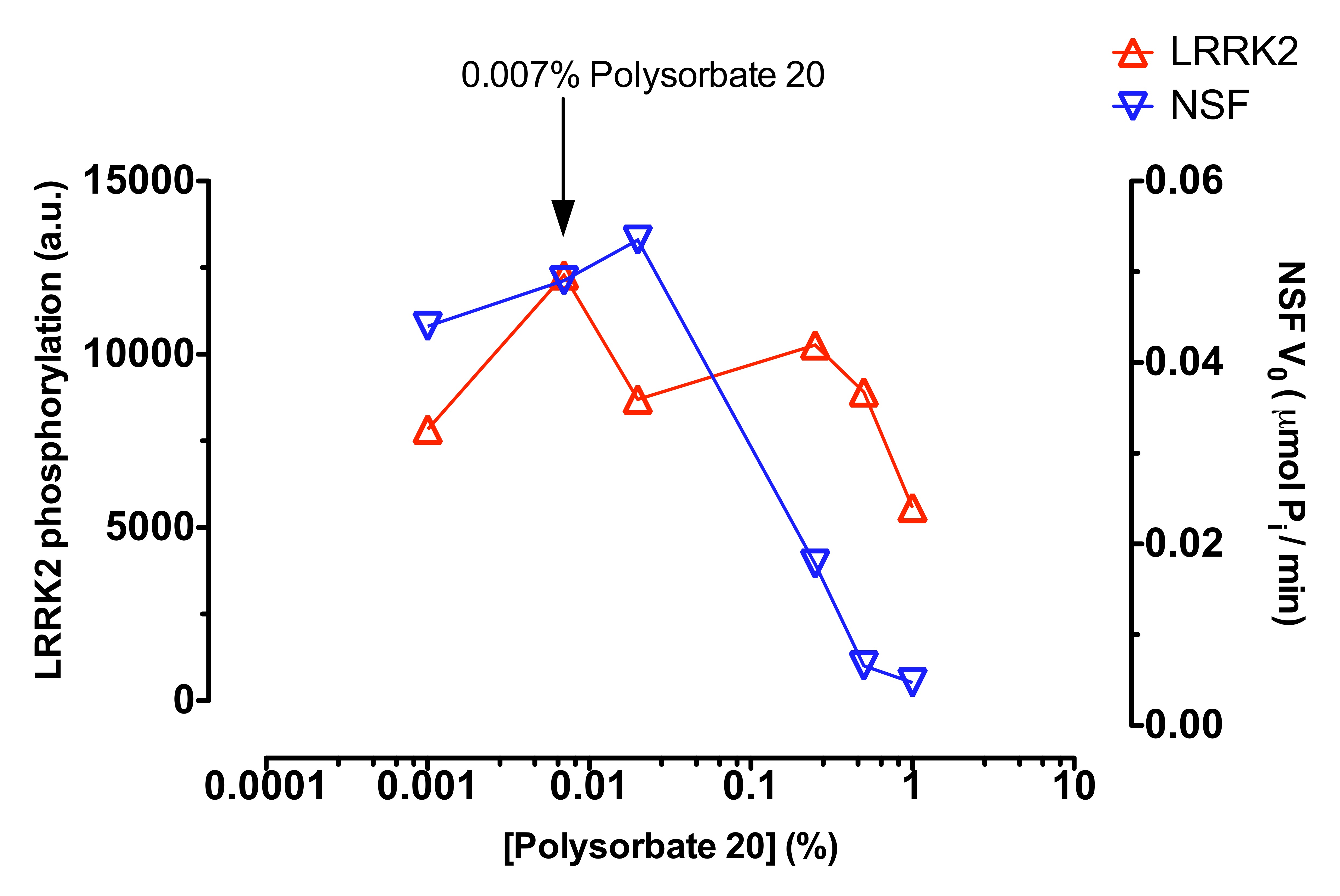


**Figure S6. Evaluation of optimal detergent concentration for LRRK2 and NSF activities.**

Optimal polysorbate 20 concentration for the reactions was evaluate within ability of LRRK2 to phosphorylate itself measuring the intensity of the band with an antibody against one of its auto-phosphorylation site (T2483). At the same time, the activity of human NSF, using the same concentration of detergent, was evaluate measuring the V_0_ of the reaction with the Malachite Green Enzyme assay.


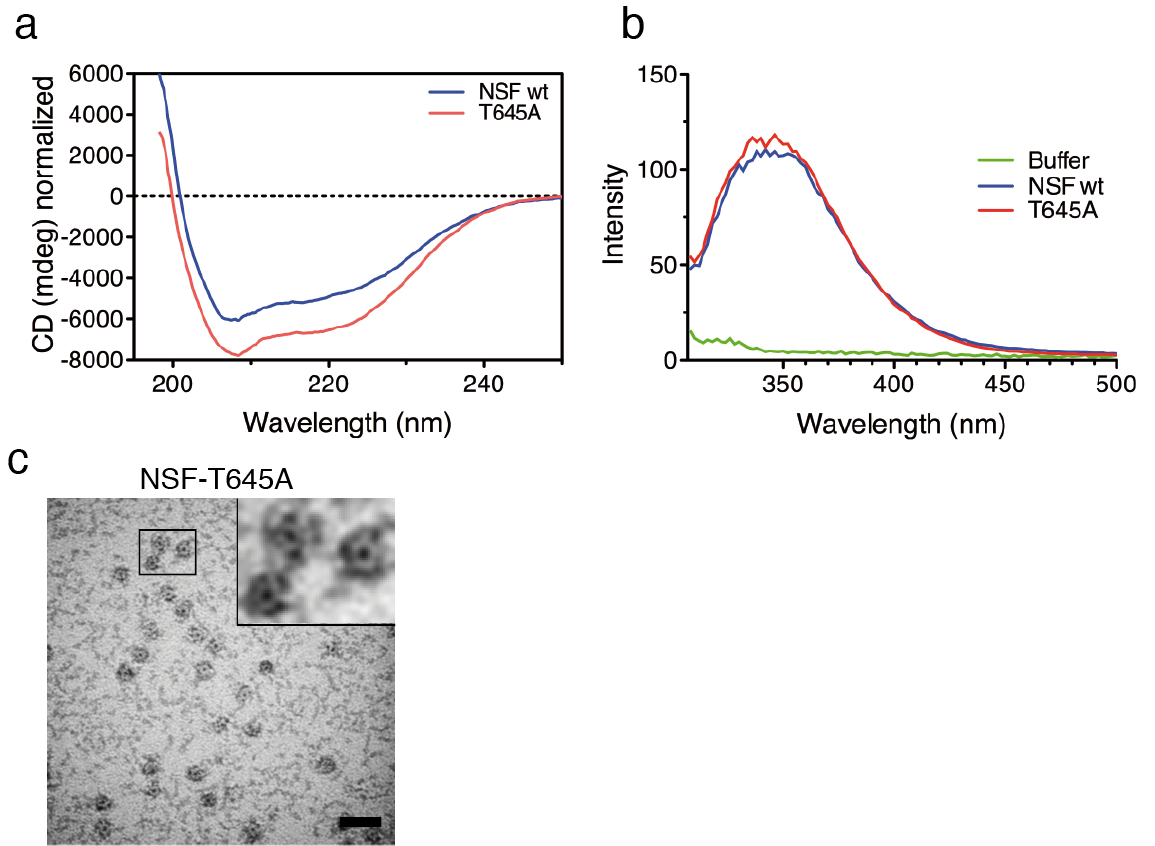


**Figure S7. NSF wild-type and T645A have similar overall folding and T645A forms hexamers.**

Recombinant human NSF wild type and NSF^T645A^ were analyzed measuring the CD and Intrinsic Fluorescence Spectra. (a) CD spectra were normalized by protein concentration previously measured with an SDS-PAGE gel. (b) The protein concentration used for Intrinsic Fluorescence measurements was 0.9 µM. (c) Representative TEM micrograph of purified Flag NSF-T645A (20 ng/µl) with 1 mM ATP and 4 mM MgCl_2_ (scale bar 50 nm).


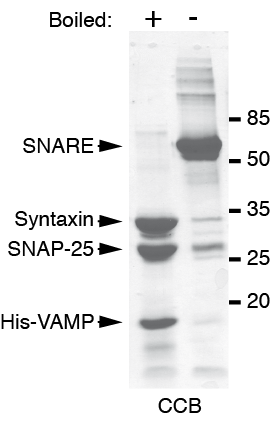


**Figure S8. Expression and purification of SDS-resistant SNARE complex.**

SDS-PAGE gel of the purified SDS-resistant SNARE complex (68 kDa) obtained by co-expression of Syntaxin, SNAP-25 and 6xHis-VAMP in *E. Coli* BL21(DE3) strain, either boiled (+) and not (-) to disassemble the three single components.
